# Evidence of absence: no relationship between behaviourally measured prediction error response and schizotypy

**DOI:** 10.1080/13546805.2017.1348289

**Published:** 2017-09-03

**Authors:** Clara S. Humpston, Lisa H. Evans, Christoph Teufel, Niklas Ihssen, David E. J. Linden

**Affiliations:** ^a^ CUBRIC, School of Psychology, Cardiff University, Cardiff, UK; ^b^ Department of Psychology, Durham University, Durham, UK; ^c^ School of Medicine, Cardiff University, Cardiff, UK

**Keywords:** Sensory prediction, associative learning, reversal learning, prediction error, psychosis continuum

## Abstract

**Introduction:** The predictive processing framework has attracted much interest in the field of schizophrenia research in recent years, with an increasing number of studies also carried out in healthy individuals with nonclinical psychosis-like experiences. The current research adopted a continuum approach to psychosis and aimed to investigate different types of prediction error responses in relation to psychometrically defined schizotypy.

**Methods:** One hundred and two healthy volunteers underwent a battery of behavioural tasks including (a) a force-matching task, (b) a Kamin blocking task, and (c) a reversal learning task together with three questionnaires measuring domains of schizotypy from different approaches.

**Results:** Neither frequentist nor Bayesian statistical methods supported the notion that alterations in prediction error responses were related to schizotypal traits in any of the three tasks.

**Conclusions:** These null results suggest that deficits in predictive processing associated with clinical states of psychosis are not always present in healthy individuals with schizotypal traits.

## Introduction

Individuals with psychosis-like experiences who are otherwise healthy and generally high-functioning are often considered to have “schizotypal traits”. Although the transition rate to frank psychosis is considered less than 50% even in individuals with clinical high risk (Fusar-Poli et al., 2012), they may nevertheless display a variety of deficits, biases, and differences in mental processes that are usually associated with clinical states. One of the most influential theories about such alterations in cognition posits that predictive processing is altered in healthy individuals prone to psychosis-like experiences (e.g., Corlett & Fletcher, 2012; Palmer, Davare, & Kilner, 2016), patients with first-episode psychosis (e.g., Corlett et al., 2007), and patients with established schizophrenia (e.g., Schlagenhauf et al., 2014).

In particular, this predictive processing model has been adduced to explain the positive symptoms of schizophrenia (delusions and hallucinations; Fletcher & Frith, 2009). This framework proposes that sensory and cognitive experiences are not simply passive events but involve the active prediction of incoming information, with the purpose of minimising prediction errors. A prediction error occurs when there is a mismatch or discrepancy between the expectation of an experience and the actual experience itself; it has been suggested that prediction errors are “a general neural coding strategy” present in the whole brain which are involved in perception, cognition, and motivational control (den Ouden, Kok, & de Lange, 2012). In the present study we tested different aspects of predictive processing, namely that in the sensory and reward domains, in relation to the same individuals’ schizotypal traits. The reward domain was further divided into associative learning and probabilistic (reversal) learning.

Sensory predictive processing is central to the monitoring and control of motor acts (Shadmehr, Smith, & Krakauer, 2010); in self-generated actions, the predicted outcome of a motor command matches the actual sensory feedback. It has been argued that this match in turn becomes our experiential marker for the sense of agency (i.e., one is the causal agent of one’s action) (Sato & Yasuda, 2005). In other words, sensory input caused by self-initiated motor acts is attenuated and there is very little prediction error to minimise (Bays, Flanagan, Wolpert, & Lackner, 2006; Brown, Adams, Parees, Edwards, & Friston, 2013). Of particular interest is the failure to assign agency to the self in delusion of control (Frith, 2012; Wilkinson, 2014), which is one of the “first-rank” symptoms of schizophrenia.

Previous studies have demonstrated sensory prediction deficits in patients with established schizophrenia (Lindner, Thier, Kircher, Haarmeier, & Leube, 2005; Shergill, Samson, Bays, Frith, & Wolpert, 2005; Synofzik, Thier, Leube, Schlotterbeck, & Lindner, 2010). To date, three studies have used a nonclinical sample with schizotypal traits who were otherwise healthy (Lemaitre, Luyat, & Lafargue, 2016; Palmer et al., 2016; Teufel, Kingdon, Ingram, Wolpert, & Fletcher, 2010). The authors of the first study (Teufel et al., 2010) found a statistically significant negative correlation between predictive processing in the sensory-motor domain (as indexed by an overcompensation score) and delusional ideation as measured by 21-item Peters et al. Delusions Inventory (PDI-21), which followed the same pattern as Shergill et al.’s (2005) finding in schizophrenia patients. Using a similar force-matching paradigm, Palmer et al. (2016) have replicated this relationship with PDI-21. Another very recent study (Lemaitre et al., 2016) used a measure of “self-tickling” as an index of sensory prediction in a student population with high and low positive schizotypy who experienced aberrant perceptions as well as passivity-like phenomena using more specific scales; it followed the same principle that self-initiated tickling sensations should be reduced due to the same sensory attenuation. The authors found that individuals who rated highly in positive schizotypy (as measured by the Schizotypal Personality Questionnaire) were better at tickling themselves, suggesting reduced sensory attenuation and therefore heightened prediction error signals.

The phenomenon of “blocking” (Kamin, 1969) in associative learning occurs when only one stimulus of a stimulus pair with a given outcome (e.g., AB+) has been previously associated with the same outcome (A+). Responses to stimulus B alone are usually attenuated (“blocked”) compared to responses to stimulus A alone or if A had not been associated with the outcome. This weakening of associative strength for B, or indeed any change in the strength of association between stimulus and outcome, can be formalised as a function of a prediction error (the Rescorla–Wagner model; see Haselgrove & Evans, 2010; Tobler, O’Doherty, Dolan, & Schultz, 2005).

There is a significant amount of evidence that in patients with schizophrenia blocking is attenuated or even absent. Patients often view both cues A and B as equally salient or equally good predictors of the outcome and the associative strength for B does not change even after previous training with A+. What is more equivocal is the particular symptom dimension that is associated with this deficit. According to the predictive processing model it would be anticipated that relationships would be found between the positive dimension of schizophrenia and a decrement in blocking. This was supported by Jones, Gray, and Hemsley (1992) who found that blocking was abolished in patients in the acute phase of the disorder, where there is a preponderance of positive symptoms, but was present in those with chronic schizophrenia. Further support is provided by Corlett et al. who found links between neural prediction error signals and delusional symptoms (2007). However, and in contrast, other researchers have found links between reductions in blocking and negative or nonparanoid symptoms (Bender, Müller, Oades, & Sartory, 2001; Moran, Al-Uzri, Watson, & Reveley, 2003; Moran, Owen, Crookes, Al-Uzri, & Reveley, 2008; Oades, Zimmermann, & Eggers, 1996).

This situation has been mirrored when researchers have adopted a continuum model of schizophrenia and examined schizotypy. Blocking has been found to be reduced in those high in: positive (Moran et al., 2003), and the negative dimension (Haselgrove & Evans, 2010), delusions (Moore, Dickinson, & Fletcher, 2011) and the distress associated with schizotypal delusion-like beliefs (Corlett & Fletcher, 2012). Given these observations both the positive and the negative dimensions of schizotypy as measured by the O-LIFE (same scale as that used by Haselgrove & Evans, 2010; Moran et al., 2003) were examined in the first instance.

Patients with schizophrenia show a multitude of deficits in reward processing (see Gold, Waltz, Prentice, Morris, & Heerey, 2008). Previous studies have used a reversal learning paradigm in both medicated and unmedicated patients (McKirdy et al., 2009; Murray, Cheng et al., 2008; Murray, Corlett et al., 2008; Reinen et al., 2016; Schlagenhauf et al., 2014; Waltz & Gold, 2007). The simplest design of a reversal learning task involves participants choosing between two visually presented stimuli (e.g., geometrical shapes): participants receive some kind of reward for choosing the correct stimulus and are punished (e.g., a reduction in the amount of money earned) for choosing the wrong stimulus. When a reversal happens, the rules are switched so that the previously correct stimulus becomes the wrong one, and vice versa.

Current evidence (e.g., Reinen et al., 2016; Schlagenhauf et al., 2014) suggest that acutely psychotic patients display an insensitivity to positive reinforcement and increased tendency to switch regardless of reversal status which corresponds to reduced error signals in the ventral striatum. In the present study, subclinical delusional ideation (as measured by PDI-21) was predicted to correlate positively with tendency to switch but negatively with perseverative behaviour. This is consistent with other studies on delusions, proneness to switching and reward insensitivity across a variety of tasks not limited to reversal learning, but also other set-shifting tasks (e.g., Cella, Dymond, & Cooper, 2009).

This study aimed to examine prediction error responses across different domains and explore the potential correlations between performance in behavioural tasks and dimensions of schizotypy as measured by various psychometric scales. The three prediction error-based tasks (force-matching, blocking, and reversal learning) were chosen because they tapped into multiple aspects of the predictive processing framework as outlined earlier and could potentially elicit error signals in different domains. Our general hypothesis was that participants scoring highly on schizotypy measures (see above for specific predictions) would exhibit deficits in prediction error responses across sensory, associative and reward domains.

## Methods

### Power calculation

Power calculations were carried out in GPower 3.1 to determine a suitable sample size. In the sensory domain, previous work examining schizotypy by Teufel et al. (2010), Lemaitre et al. (2016), and Palmer et al. (2016) indicate effect sizes ranging from 0.35 to 0.58. Given an alpha level of .05 and a power level of 0.90, this gives an estimated sample size of 23–78 (two-tailed correlations). For the blocking task, previous studies by Haselgrove and Evans (2010) and Moran et al. (2003) have estimated effect sizes from 0.30 to 0.39, giving a sample size of 61–109. In the reward domain due to a lack of studies examining schizotypy, an effect size of 0.59 has been generated from schizophrenia patient datasets (Schlagenhauf et al., 2014) yielding a sample size of 22. However, it should be noted that the effects in schizotypy would be anticipated to be smaller and hence a larger sample size would be necessary. In order to maximise power, we decided to recruit up to 120 participants (greater than the highest number estimated).

### Participants

One hundred and fifteen healthy volunteers from across Cardiff University (mainly undergraduate students, but also postgraduates and staff) were recruited through the Experimental Management System and the university’s electronic Noticeboard system. All participants gave written informed consent and were fully debriefed after the experimental session, and received either course credits or a single sum of £15 after the session as reimbursement for their time. The study was approved by the School of Psychology Research Ethics Committee.

Thirteen participants were excluded from the current study because they failed to meet the inclusion criteria for one or more of the behavioural tasks (see below for the specific criteria and the number excluded for each task). The final 102 participants consisted of 21 males and 81 females with a mean age of 21.96 (SD = 3.14) years. Assuming the smallest effect size of 0.30, this has yielded an achieved power of 0.88.

### Procedure

The three tasks reported in the current paper were a part of a larger battery consisting of five tasks (also including action and verbal source monitoring tasks which are reported separately—Humpston, Linden, & Evans, 2017); each experimental session took two hours in total. Participants were all tested individually.

### Force-matching task

This procedure (adapted from Teufel et al., 2010) focused on the sensory type of prediction error. Participants were asked to place their left index finger under a lever attached to a torque motor which then applied four different levels of forces in a random order. Participants were asked to match the presented force in two conditions, which were counterbalanced across participants. In the “Finger” condition, participants matched the force by directly pressing down onto the tip of the lever using their right index finger. In the “Slider” condition, participants matched the force indirectly by moving a linear potentiometer up and down which controlled the torque motor. The gain of the slider was 0.5 N/cm. Participants received training of the task in the form of a practice session (8 trials) of both conditions before progressing to testing sessions of 32 trials each. Five participants were excluded on this task because the differences in applied forces deviated more than two standard deviations from the mean, which was the same criterion used by Teufel et al. (2010).

### Kamin blocking task

This associative learning task used the same paradigm as that by Haselgrove and Evans (2010, Study 1). Participants were asked to play the role of a hospital inspector and evaluate whether certain food items and pairings of food items caused food poisoning by entering numbers with the keyboard between 1 (completely safe to eat) and 9 (completely dangerous) with number 5 as being uncertain. As apparent from the task design in Table 1, if there is blocking present the participants’ ratings of B would be smaller than those for D or F; in other words, blocking occurs because the associative strength for B from compound AB+ is attenuated due to prior association with stimulus A+. Data from nine participants (with one meeting neither the inclusion criteria for blocking nor force-matching) were excluded due to a failure to learn stimulus-outcome associations in Stage 1 and/or 2, or failure to respond with appropriate keys (i.e., pressing the same keys no matter what the trial was). This exclusion criterion was the same as that used by Haselgrove and Evans (2010). The blocking effect was denoted as a final rating of D minus B.

**Table 1.** Design of Kamin blocking task.

Stage 1	Stage 2	Test
A+	AB+	B
	CD+	D
E−	EF+	F
	K+	K
GH+	L−	
IJ−	IJ−	

Notes: Cues A to L indicate each food item, either associated with the outcome of food poisoning (+) or not (−). GH+. L− and IJ− are filler trials.

### Reversal learning task

This task aimed to tap into the reward/motivational type of prediction error and was identical to that described in Lancaster et al. (2015) and the “private condition” in Ihssen, Mussweiler, and Linden (2016). Participants were asked to choose between two coloured squares, blue and green, which were displayed on the same screen side by side. Participants earned 1 penny (reward; +1p) if they chose the correct colour and lost 1 penny (punishment; −1p) if they chose the wrong colour.

At the beginning the colour blue was set to be the correct colour; however, after a variable number (between 7 and 15) of trials the reward/punishment contingencies were reversed (true reversal) so that blue became the wrong colour and was punished, whereas green became the corrected colour and was rewarded. Feedback was given immediately after each choice in the form of a smiley face (as a sign of winning money) or a sad face (as a sign of losing money). Probabilistic errors were also included between two true reversal trials, whose numbers were again variable (between 1 and 3). Such errors meant that participants were unexpectedly punished even though they chose the correct colour (i.e., “wrong feedback”). Participants were told that only one colour would be correct at one time and were aware of the existence of true reversals as well as probabilistic error trials, but did not know when they would occur. The task contained 132 trials with an average of 11 true reversals in total. No participants were excluded on the basis of performance on this task.

### Questionnaires

Participants completed three questionnaires on the different dimensions of schizotypy: the Oxford-Liverpool Inventory of Feelings and Experiences (O-LIFE; Mason, Claridge, & Jackson, 1995), the PDI-21 (Peters, Joseph, Day, & Garety, 2004), and the Cardiff Anomalous Perceptions Scale (CAPS; Bell, Halligan, & Ellis, 2006). We used three schizotypy scales because they have different emphases: the O-LIFE is a multidimensional tool which allows the researcher to assess feelings and experiences that are akin to the positive and negative dimensions of schizophrenia, whereas the other two scales measure specific experiences: delusional ideation with the PDI-21 and perceptual disturbances/hallucinations on the CAPS. These scales have been examined in relation to the various types of prediction error and so were included in this study to allow us to fully replicate previous study procedures.

### Analysis of behavioural data

We employed a parallel analysis strategy in which both frequentist (Null Hypothesis Significance Testing) and Bayesian approaches were used; Bayes factors were calculated to explore the strength of evidence or the confidence with which the null hypotheses are supported. It has been suggested that Bayesian approaches are resistant to multiple comparison problems (Dienes, 2011). All frequentist data analyses were carried out using SPSS 23 (IBM Corp.) and all correlations were two-tailed; all Bayesian analyses (Bayesian Correlation Pairs) were carried out in JASP Version 0.8.0.0 (https://jasp-stats.org/).

Consistent with previous studies (Haselgrove & Evans, 2010; Schlagenhauf et al., 2014; Teufel et al., 2010), measures of prediction error-based behavioural responses are as follows: in the force-matching task, an overcompensation score was calculated for each participant by subtracting the mean difference between active (applied by the participant) and passive (original force applied by the machinery) forces in the Slider condition from that in the Finger condition. In the Kamin blocking task, the extent of blocking was calculated by the final rating for cue D minus the final rating for cue B. Participants’ ratings for each learning stage are plotted as line graphs to ensure that learning occurred. Total accuracy, post-probabilistic error accuracy (an index of switching or “switchiness”) and post-true reversal accuracy (an index of perseveration) were entered in the analysis as dependent variables for the reversal learning task.

For all three tasks, the main outcome measures were correlated with corresponding schizotypy scales (the same as those used in frequentist statistics) by using a Bayesian Correlation Pairs analysis in JASP. For the force-matching task, this was the overcompensation score and the total score of PDI-21; for Kamin blocking, this was the blocking score and the unusual experiences score of O-LIFE as well as PDI-21 distress subscale; and for the reversal learning task the correlation was done between switching score and PDI-21 total score. Bayesian factors in the form of BF_01_ (null over alternative) were calculated from a priori hypotheses regarding the direction of the correlation together with robustness checks to reflect the strength of evidence. In the cases of force-matching and Kamin blocking, the direction of the correlations was hypothesised to be negative whereas for reversal learning, the direction of the correlation was hypothesised to be positive. Beta* (stretched beta) prior width for these correlations was set to a relatively conservative value of 0.5.

## Results

### Schizotypy questionnaire scores

Descriptive data for the three scales completed by the remaining 102 participants included in the current study are shown in Table 2. Normative means taken from the original scales (PDI-21 from Peters et al., 2004; CAPS from Bell et al., 2006; and O-LIFE from Mason & Claridge, 2006) were also included for comparison.

**Table 2.** Descriptive data for schizotypy scales and their subscales (*N* = 102).

	Mean (SD)	Range	Normative mean (SD)
PDI-21 total Y/N	5.88 (3.47)	0–16	6.7 (4.4)
PDI-21 distress	15.95 (12.01)	0–51	15.5 (14.1)
PDI-21 preoccupation	14.84 (11.45)	0–57	15.4 (14.1)
PDI-21 conviction	17.92 (11.79)	0–52	20.4 (16.0)
CAPS total Y/N	8.29 (6.03)	0–22	7.3 (5.8)
CAPS distress	20.92 (18.13)	0–84	15.5 (14.5)
CAPS intrusiveness	22.43 (19.09)	0–92	18.0 (17.0)
CAPS frequency	17.65 (15.72)	0–79	14.6 (14.2)
O-LIFE UnExp	7.14 (5.44)	0–25	8.82 (6.16)
O-LIFE IntAn	4.79 (4.31)	0–22	6.38 (4.49)

Notes: SD: standard deviation; PDI-21: 21-item Peters et al. Delusions Inventory; CAPS: Cardiff Anomalous Perceptions Scale; O-LIFE: Oxford-Liverpool Inventory of Feelings and Experiences; UnExp: unusual experiences; IntAn: introvertive anhedonia.

### Force-matching

**Figure 1.**
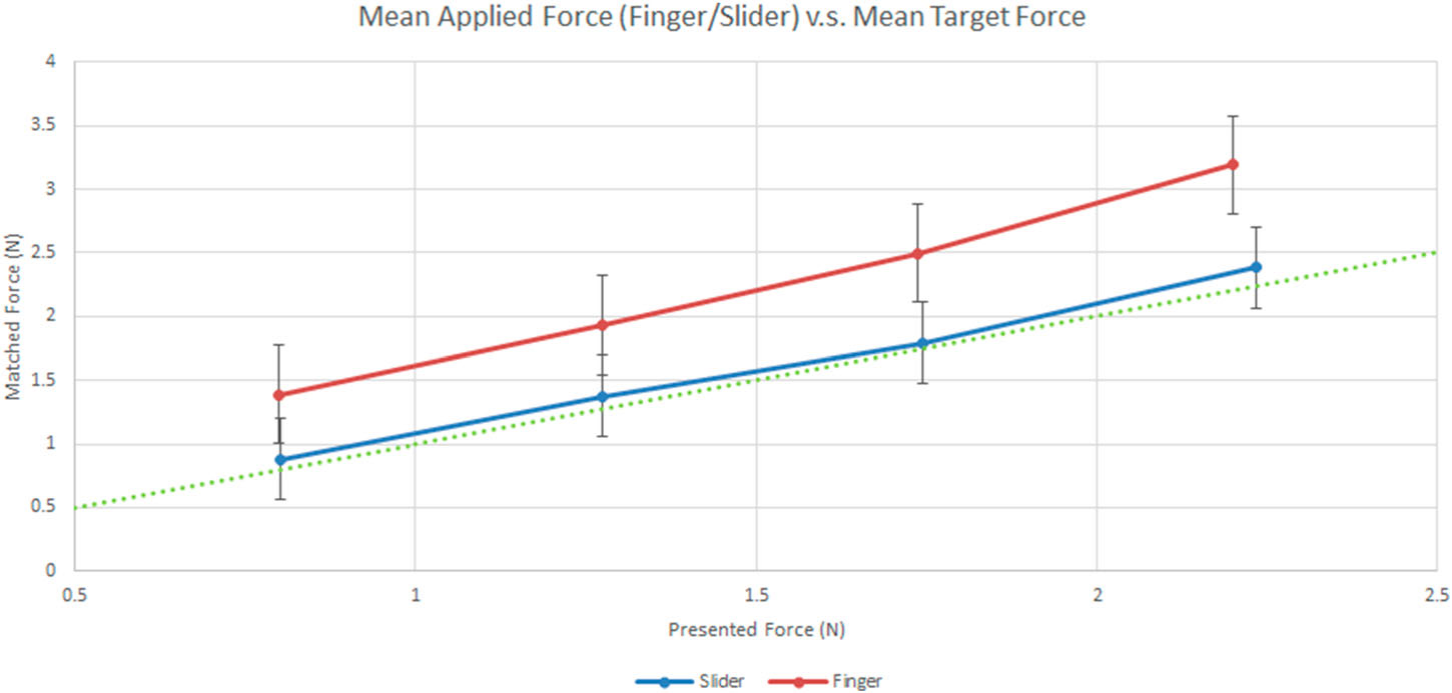
Comparison between mean difference of active and passive forces in the Finger and Slider conditions. The dotted line indicates perfect performance.

Participants consistently applied more force in the Finger than in the Slider condition, demonstrating the overcompensation effect (Figure 1). A paired-sample *t*-test showed that the mean difference between active and passive forces applied in the Finger condition was significantly greater than that in the Slider condition [*t*(101) = 13.26, *p* < .001)].

In terms of the relationship between the overcompensation score and delusional ideation (as measured by PDI-21 total score), a Spearman’s correlation was calculated. We found a non-significant correlation between these two variables [*ρ*(100) = .139, *p* = .163]. Furthermore, no significant relationships were found between the overcompensation score and any of the other schizotypy questionnaires (see Table 3).

**Table 3.** Nonparametric bivariate correlation coefficients (Spearman’s rho, two-tailed) between schizotypy measures and task measures (*N* = 102).

	PDI-21Tot	PDI-21Dis	PDI-21Con	PDI-21Pre	CAPS Tot	CAPS Dis	CAPS Int	CAPS Fre	O-LIFE UnExp	O-LIFE IntAn
Force-matching overcompensation	.139	.143	.186	.166	.074	.085	.073	.109	.127	.100
Blocking score	.108	.130	.132	.128	.130	.126	.123	.136	.028	−.196
Post-reversal perseveration	.025	.032	−.010	.012	−.113	−.086	−.093	−.104	−.075	.013
Post-probabilistic error switching	.008	.075	.015	−.013	.063	.071	.064	.026	.089	.046

Notes: PDI-21: 21-item Peters et al. Delusions Inventory; Tot: total yes/no endorsements; Dis: distress; Con: conviction; Pre: preoccupation; CAPS: Cardiff Anomalous Perceptions Scale; Int: intrusiveness; Fre: frequency; O-LIFE: Oxford-Liverpool Inventory of Feelings and Experiences; UnExp: unusual experiences; IntAn: introvertive anhedonia. None of these relationships reached conventional levels of significance (*p* > .05) even without the application of a correlation for multiple comparisons.

### Kamin blocking

**Figure 2.**
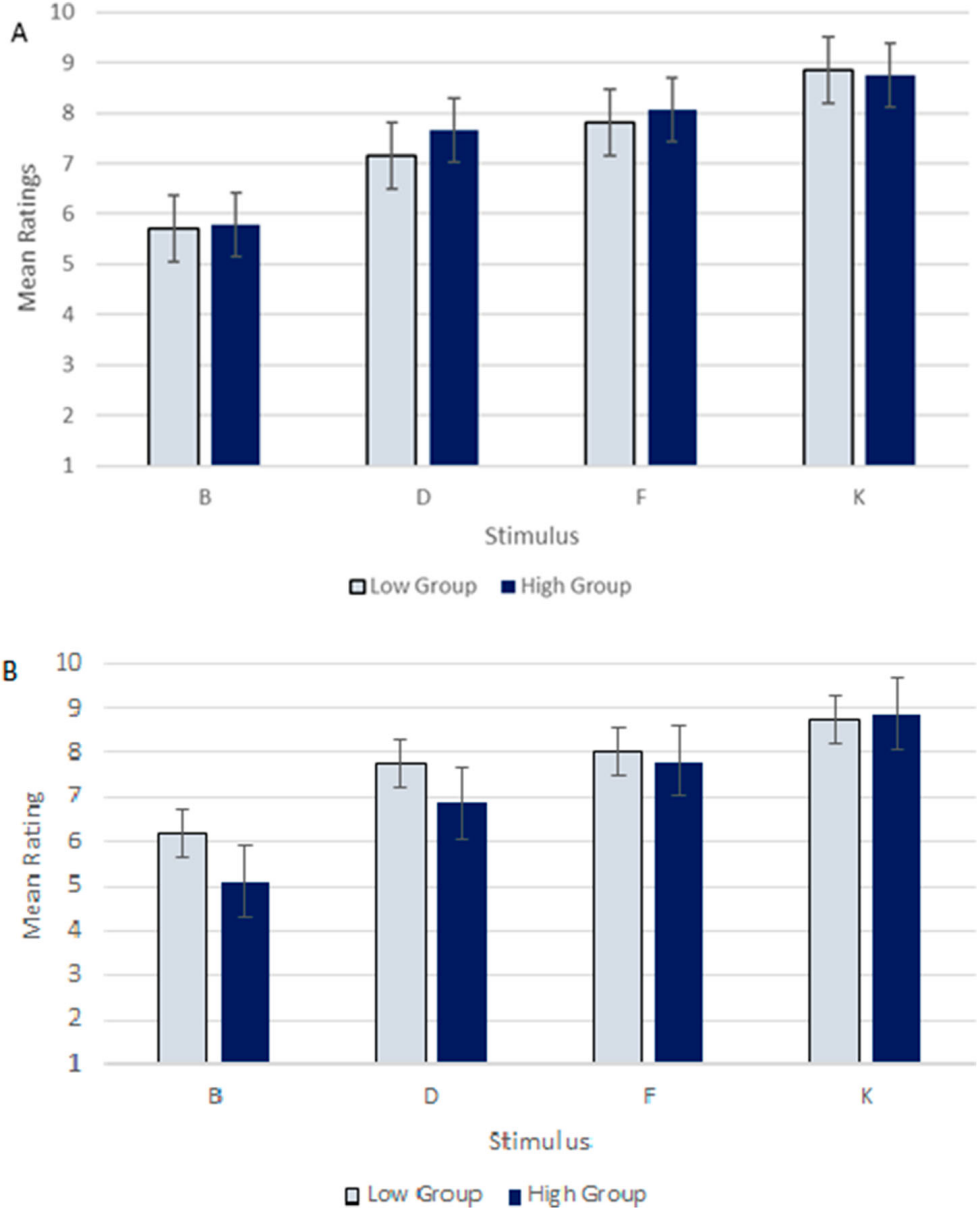
Low and high group ratings for each stimulus in the test stage for O-LIFE unusual experiences (A) and introvertive anhedonia (B).

When correlated with O-LIFE unusual experience and introvertive anhedonia (positive and negative schizotypy, respectively) scores, we found no significant correlation between these variables (*ρ*(100) = .028, *p* = .782 for the positive dimension and *ρ*(100) = −.106, *p* = .290 for the negative dimension). In order to replicate the methodology used by Haselgrove and Evans (2010), median splits of O-LIFE unusual experiences and introvertive anhedonia scores were computed which divided the participants into high and low positive/negative schizotypy groups. The median value for unusual experiences was 6, with scores equal to these values included in the “low” group whereas that for introvertive anhedonia was 4. Figure 2 visualises the mean ratings for each stimulus in the testing stage for low and high positive (A) and negative (B) schizotypy groups. There was no significant effect of group for either positive [*F*(1, 416) = 3.544, *p* = .680] or negative [*F*(1, 416) = 15.975, *p* = .078] schizotypy. Table 3 shows that all correlations with other schizotypy subscales were also non-significant.

**Figure 3.**
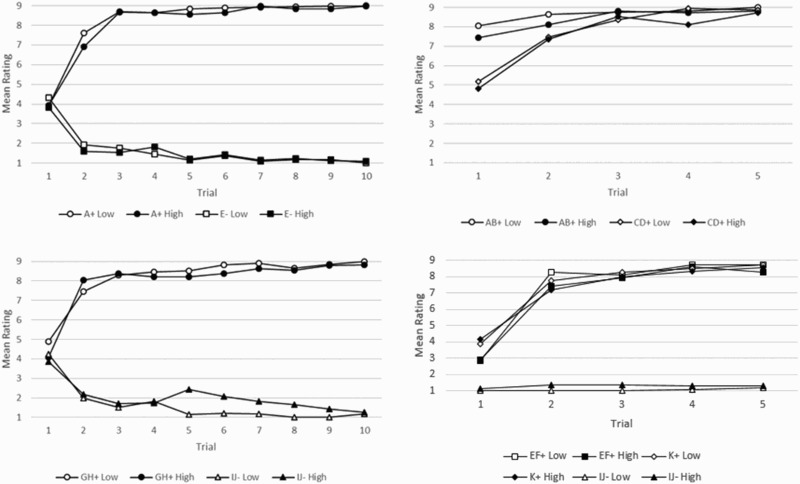
Low and high group ratings for unusual experiences across learning stages. + and − refer to the presence or the absence of the outcome, respectively.

Further still, we investigated the learning of stimulus-outcome associations in Stages 1 and 2 to determine whether there were differences between individuals high and low in positive and negative schizotypy. There were no differences in the learning acquisition between these two groups in unusual experiences (Figure 3). For Stage 1, a two-way analysis of variance (ANOVA) with factors of group (high versus low unusual experiences) and stimulus (A+ and E−) and mean ratings as dependent variable yielded a highly significant effect of stimulus [*F*(1, 200) = 4013.03, *p* < .001], but no significant effect of group [*F*(1, 200) = 0.715, *p* = .399] or group*stimulus interaction [*F*(1, 200) = 0.372, *p* = .543]. An identical ANOVA carried out with stimuli GH+ and IJ− also revealed a highly significant effect of stimulus [*F*(1, 200) = 1980.24, *p* < .001], no significant effect of group [*F*(1, 200) = 0.688, *p* = .408] but a significant group*stimulus interaction [*F*(1, 208) = 4.398, *p* = .037].

**Figure 4.**
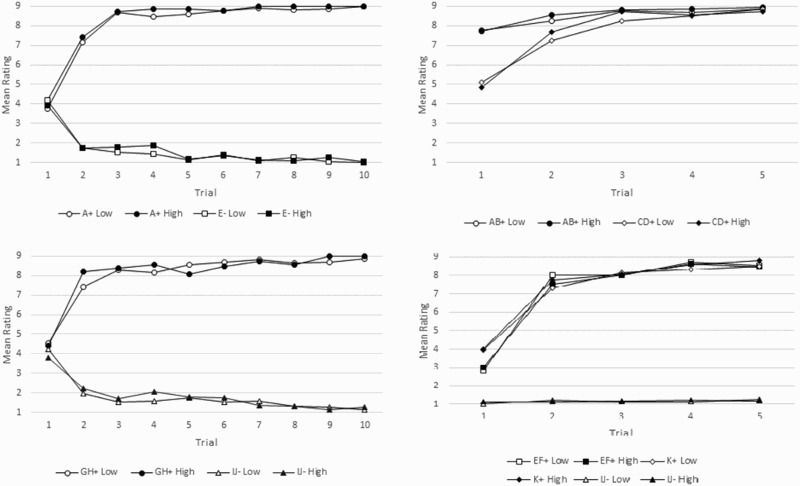
Low and high group ratings for introvertive anhedonia cross learning stages. + and − refer to the presence or the absence of the outcome, respectively.

For Stage 2, a two-way ANOVA performed with factors of group (high/low unusual experiences) and stimulus (AB+, CD+) and mean ratings as dependent variable did yield a weak but significant effect of group [*F*(1, 200) = 4.668, *p* = .032], a highly significant effect of stimulus [*F*(1, 200) = 41.904, *p* < .001], but no significant interaction [*F*(1, 200) = 0.012, *p* = .911]. An identical ANOVA carried out with stimuli EF+, K+, and IJ− also revealed a highly significant effect of stimulus [*F*(2, 300) = 968.28, *p* < .001], no significant effect of group [*F*(1, 300) = 0.426, *p* = .514] and no significant group*stimulus interaction [*F*(2, 312) = 1.765, *p* = .173]. We found a similar pattern of results when identical ANOVAs were performed with data split by negative schizotypy scores (as measured by introvertive anhedonia, Figure 4): only stimulus type yielded significant effects whereas group status did not.

### Reversal learning

**Figure 5.**
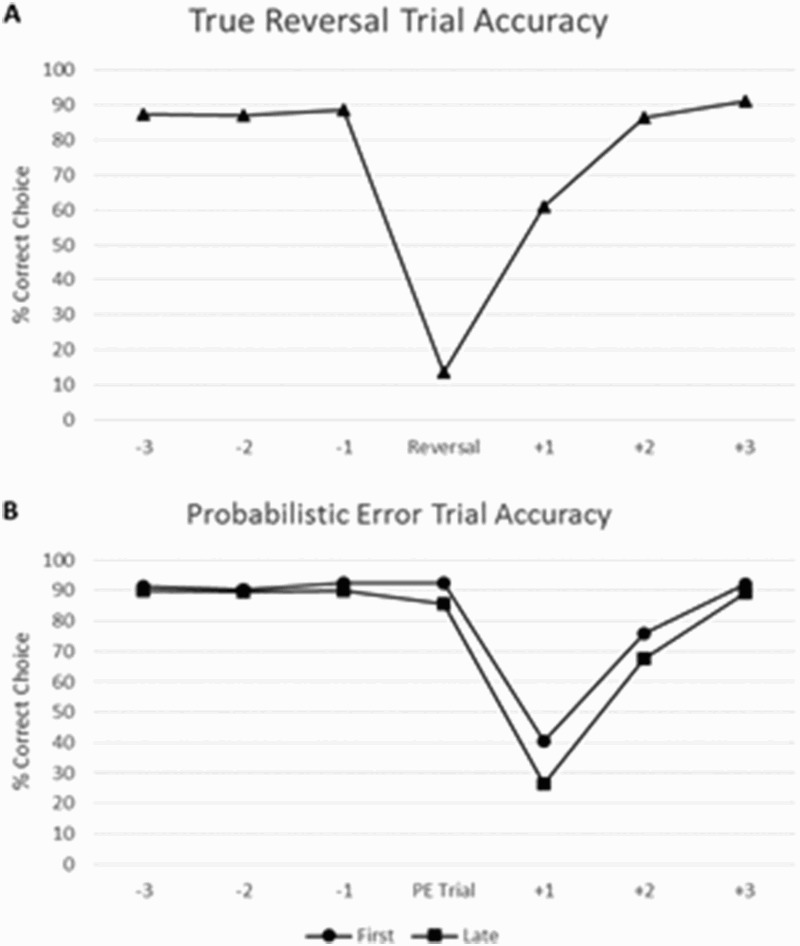
Accuracies of true reversal trials (A) and probabilistic error trials (B).

Figure 5 shows mean accuracy data for trials surrounding true reversals and probabilistic errors; the latter was further divided into first and late (second/third) probabilistic errors. Accuracy was greatly reduced at reversal trials from 90% to below 10% and then recovered within two trials to the pre-reversal level. Trials after late probabilistic errors demonstrated a lower accuracy than those after the first error (30% versus 40%). It required at least two further trials to restore task performance back to ceiling level in both situations. This pattern of results is compatible with other studies employing this and similar reversal learning paradigms (Ihssen et al., 2016). Switching and perseveration scores for each participant were calculated as the inverse of post-probabilistic error and post-reversal accuracies. In a subsequent correlational analysis switching score was not significantly correlated with delusional ideation as measured by PDI-21 total scores [*ρ*(100) = .008, *p* = .937] and neither was perseveration [*ρ*(100) = .025, *p* = .806). Table 3 shows further non-significant correlations with other schizotypy measures.

### Bayesian Correlation Pairs

**Figure 6.**
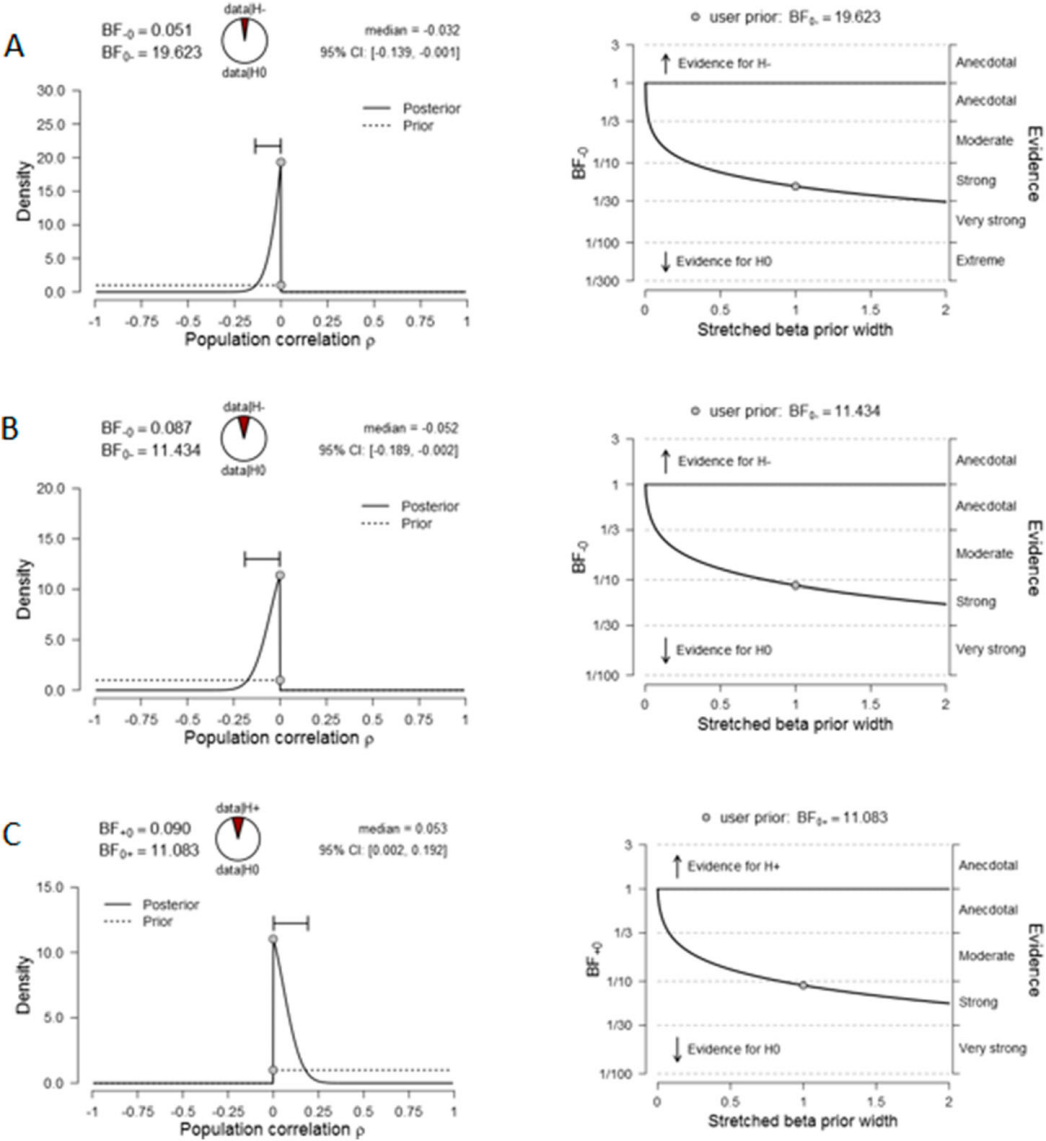
Results from Bayesian Correlation Pairs analyses. Panels A, B and C show results for the force-matching, Kamin blocking and reversal learning tasks, respectively. CI: credibility interval; BF: Bayes factor.

Results from Bayesian analyses are presented in Figure 6. For the force-matching task, BF_01_ was estimated to be 19.623, meaning that the data provided were highly in favour of the null hypothesis (19 times the likelihood of the alternative hypothesis, in this case a negative correlation between overcompensation and PDI-21 total scores) with strong to very strong evidence, meaning that there was a significant amount of support for no effect.

For the Kamin blocking task where BF_01_ was estimated to be 11.434 for the positive dimension, which also meant that the data provided support in favour of the null hypothesis (10 times the likelihood of the alternative hypothesis, in this case a negative correlation between blocking and O-LIFE unusual experiences scores). BF_01_ was estimated to be 5.092 for the negative dimension, which also meant that the data provided support in favour of the null hypothesis (5 times the likelihood of the alternative hypothesis, in this case a negative correlation between blocking and O-LIFE introvertive anhedonia scores, graphs not included in figure). Robustness checks demonstrated a moderate to strong level of evidence favouring the null hypothesis for both correlations.

For switching tendency of the reversal learning task, BF_01_ was estimated to be 11.083 which meant that the data was favouring the null hypothesis 11 times; in other words, the alternative hypothesis was highly improbable. Robustness checks demonstrated a strong level of evidence for the null hypothesis. In terms of the correlation between perseveration and PDI-21 total score, BF_01_ values were estimated to be 8.031, which favoured the null hypothesis with strong to very strong levels of evidence (graphs not included in Figure 6).

## Discussion

The current study investigated the relationships between different types of predictive processing and domains of psychometrically defined schizotypy in the same individuals. We did not find evidence for disrupted sensory predictive processing (as indexed by the force-matching task) in individuals with high scores of delusional ideation. Moreover, there was no significant difference in associative learning (as indexed by the blocking effect) between individuals with high and low positive or negative schizotypy or distress caused by delusion-like beliefs. Also, there was no evidence for alterations in switching tendency or perseveration as indexed by the reversal learning task in individuals with higher levels of delusional ideation. Importantly, our study failed to demonstrate the same pattern of findings from previous studies which separately investigated sensory prediction, blocking phenomenon and reversal learning in relation to domains of schizotypy.

In the force-matching task, participants significantly overcompensated in the finger condition, which demonstrates the classic force-matching effect, which has been found in all (?) previous studies. However sensory attenuation was not impaired in individuals with high delusional ideation. The use of PDI-21 rather than the PDI-40 may raise some concerns about the omission of items capturing delusions of control or passivity-like experiences which, by definition, have higher relevance with sensory prediction than other delusions such as paranoia. This is supported by the observation that in addition to positive schizotypy in general, Lemaitre et al. (2016) also found a significant negative correlation specifically between passivity-like experiences and the index of sensory attenuation. However, given that previous studies examining force-matching, such as those by Teufel et al. (2010) and Palmer et al. (2016), used the PDI-21 and not the PDI-40, this does not explain why we failed to observe this relationship in the current study. In addition, the PDI-21 was derived from the 40-item version with very similar psychometric properties (Peters et al., 2004). One methodological detail which differs between the current study and that of Teufel et al. (2010) is that in the latter study more repetitions were used to average applied and presented force (eight rather than four levels of forces). Therefore, it may be the case that the measurements were somewhat noisier in the current study because of the necessity of reducing the length of tasks to accommodate for the overall duration of testing (two hours).

In the associative learning task, the blocking task we utilised was exactly the same as that used by Haselgrove and Evans (2010). In contrast to their study, we failed to find any relationships with the negative dimension of schizotypy, even when we followed the same analytic methods used in that study (e.g., carrying out a median split with the same median). Given our well-powered study it could be that the failure to find this relationship might have been affected by other factors such as smoking status which was not measured in the current study. For example, nicotine has been shown to reduce dopamine release (Zhang & Sulzer, 2004) and may attenuate the prediction error responses mediated by dopamine. Furthermore, we also did not find any significant relationships between blocking and any other schizotypy dimensions, such as the positive dimension as previously found by Moran et al. (2003), the total PDI score (as found by Moore et al., 2011) or the distress aspect of delusional ideation (as found by Corlett & Fletcher, 2012). For these correlations we used the same schizotypy measures as what previous studies used but the blocking task and measure of this phenomenon were different. For example, Corlett et al. used computer-paced tasks whereas we used a self-paced task, and the former group did not use behavioural measures for blocking unlike in our study. There is some debate about whether prediction error as a latent process in associative learning is best studied by neuroimaging or behavioural methods, or perhaps a combination of both (see Corlett & Fletcher, 2015; Griffiths, Langdon, Le Pelley, & Coltheart, 2014).

In the reversal learning task, we used switching tendency as an index of reward sensitivity driven by prediction error-related learning and found no significant associations between an increased tendency to switch after probabilistic errors and delusional ideation in either frequentist or Bayesian statistical analyses. In fact, accurate responding was restored very soon after both true reversals and probabilistic errors, suggesting that participants performed the task effectively and learnt when to switch or stay relatively quickly. These findings are clearly in contrast with findings in schizophrenia (e.g., Schlagenhauf et al., 2014), but due to a lack of studies using reversal learning in healthy schizotypy, comparisons can only be made with other set-shifting tasks in individuals prone to psychosis-like experiences (e.g., Cella et al., 2009) which once again do not support current findings.

Our hypotheses focused on delusion-proneness and we did not find significant correlations between hallucination measures (i.e., CAPS) and behavioural performance in the current study. Hallucinations have been recently linked with predictive coding (e.g., Horga, Schatz, Abi-Dargham, & Peterson, 2014) in established schizophrenia; however, in nonclinical groups hallucinations can also persist without causing distress or leading to a need for psychiatric care (Hill, Varese, Jackson, & Linden, 2012; Johns et al., 2014; Linden et al., 2010) in many high-functioning individuals.

In our sample, participants were all functioning relatively highly. In fact, although there were some individuals who endorsed the more “bizarre” items such as thought echo in the schizotypy questionnaires, these were a very small minority of participants. The majority of schizotypy scores in our sample were positively skewed towards “normal experience” even though the means of these scores were comparable to those from previous general population studies of schizotypal traits.

However, it is also possible that there was potential disconnection between subjective experiences of schizotypy and objective measures of neurocognitive deficits, in which the subjective complaints from psychometrically measured schizotypy do not match the magnitude of deficits seen in behavioural tasks (e.g., Chun, Minor, & Cohen, 2013).

Cross-sectional studies of this kind are unable to establish causal relationships. A possibility for future research would thus be a longitudinal study with structured assessments at regular intervals in order to determine the persistence of psychosis-like experiences and any rate of transition to clinical disorders, as well as incorporating a range of methods for measuring prediction error responses (e.g., combining imaging with behavioural testing).

Our study may also have been affected by a selection bias where only participants with certain traits and interests were “attracted” to research or motivated to take part in the study (see Martin et al., 2016, who found significant relationships between non-participation and individuals’ risk factors for schizophrenia) which would further reduce the generalisability of these findings. However, this factor would similarly apply to previous studies of this topic.

In sum, although much caution needs to be taken when interpreting the results, the present study furthers our understanding of the construct of schizotypy by employing an integrative approach to predictive processing in relation to different domains of schizotypal traits in a large sample of high-functioning individuals with no past or present psychiatric diagnosis. Our null findings suggest that predictive processing mechanisms, at least in the forms of sensory, associative and reward prediction error responses, are *not* always associated with positive schizotypal personality traits in the general population.
